# Pressure-induced enhancement in the thermoelectric properties of monolayer and bilayer SnSe_2_

**DOI:** 10.1098/rsos.171827

**Published:** 2018-03-28

**Authors:** Daifeng Zou, Chuanbin Yu, Yuhao Li, Yun Ou, Yongyi Gao

**Affiliations:** 1 School of Physics and Electronic Science, Hunan University of Science and Technology, Xiangtan 411201, People's Republic of China; 2 Shenzhen Key Laboratory of Nanobiomechanics, Shenzhen Institutes of Advanced Technology, Chinese Academy of Sciences, Shenzhen 518055, People's Republic of China; 3 State Key Laboratory of Mechanics and Control of Mechanical Structures, Nanjing University of Aeronautics and Astronautics, Nanjing 210016, People's Republic of China

**Keywords:** thermoelectric, SnSe_2_, first-principles, high pressure

## Abstract

The electronic structures of monolayer and bilayer SnSe_2_ under pressure were investigated by using first-principles calculations including van der Waals interactions. For monolayer SnSe_2_, the variation of electronic structure under pressure is controlled by pressure-dependent lattice parameters. For bilayer SnSe_2_, the changes in electronic structure under pressure are dominated by intralayer and interlayer atomic interactions. The *n*-type thermoelectric properties of monolayer and bilayer SnSe_2_ under pressure were calculated on the basis of the semi-classical Boltzmann transport theory. It was found that the electrical conductivity of monolayer and bilayer SnSe_2_ can be enhanced under pressure, and such dependence can be attributed to the pressure-induced changes of the Se–Sn antibonding states in conduction band. Finally, the doping dependence of power factors of *n*-type monolayer and bilayer SnSe_2_ at three different pressures were estimated, and the results unveiled that thermoelectric performance of *n*-type monolayer and bilayer SnSe_2_ can be improved by applying external pressure. This study benefits to understand the nature of the transport properties for monolayer and bilayer SnSe_2_ under pressure, and it offers valuable insight for designing high-performance thermoelectric few-layered SnSe_2_ through strain engineering induced by external pressure.

## 1. Introduction

Thermoelectric materials, which can directly and reversibly convert heat into electricity, have potential applications in power generation and refrigeration [1]. The conversion efficiency of thermoelectric materials is described by the figure of merit, *ZT*, which is defined as *ZT* *=* *S*^2^*σT/*(*κ*_e_*+κ*_l_), where *S*, *σ, κ*_e_ and *κ*_l_ are the Seebeck coefficient, electrical conductivity, electric and lattice thermal conductivities, respectively. A good thermoelectric material must possess high *ZT* value. The current researches are focused on enhancing the *ZT* value by increasing the Seebeck coefficient and electrical conductivity values while reducing the value of thermal conductivity. However, this is not easy because the correlation and coupling among Seebeck coefficient *S*, electrical conductivity *σ* and electric thermal conductivity *κ*_e_ make it extremely difficult to control independently, and the parameter of lattice thermal conductivity *κ*_l_ is related to crystal structure that is difficult to manipulate as well. As a result, most of the recent efforts in *ZT* improvement have been concentrated around these compounds that possess intrinsically low lattice thermal conductivity and then optimize electronic properties *S^2^σ* (power factor: PF).

Because of their quantum confinement effect, two-dimensional materials such as metal dichalcogenides have recently gained attention due to anharmonicity leading to low thermal conductivities and high figures of merit [2,3]. For example, the highest dimensionless figure of merit *ZT* of monolayer WSe_2_ can reach 0.8 at 1200 K [4]. Moreover, it was reported that a maximum *ZT* of 1.65 was achieved at 300 K in ZrS_2_ monolayer due to the *κ*_l_ is 3.29 W K^−1^m^−1^ [5]. Recently, the binary compound SnSe_2_ is predicted as a promising thermoelectric material based on it possessing comparative *β* value with that of SnSe [6]. Based on the naturally layered structure which can possess very low lattice thermal conductivity, the two-dimensional SnSe_2_ compound is believed to be a potential candidate for thermoelectric materials. As a member of the metal dichalcogenides, SnSe_2_ is a hexagonal close-packed CdI_2_-type structure, and it consists of alternating sandwiched sub-layers bonded by van der Waals (vdW) interaction [7]. Ding *et al.* [8] studied the thermoelectric properties of *n*-type SnSe_2_ and the calculated optimal *ZT* of 2.95 in the *a* direction at 800 K was observed due to the strong anisotropic feature. Just like the other metal dichalcogenides, SnSe_2_ can be easy to construct two-dimensional sheets due to the weak interlayer bonding [9]. On the theoretical side, electronic and magnetic properties of SnSe_2_ nanostructures under strains were calculated using first-principles calculations, and the controllable electronic and magnetic properties were presented [10]. Li *et al*. [11] use thermoelectric properties of SnSe_2_ monolayer by using first-principles methods combined with Boltzmann transport theory, showing that SnSe_2_ monolayer is a promising candidate for thermoelectric applications.

Applying variable pressure is proved to be an effective tool to tune the electronic and thermoelectric properties in some thermoelectric semiconductors [12]. The thermoelectric transport properties of these bulk compounds, such as SnSe [13], BiCuOSe [14], Bi_2_Te_3_ [15], PbTe [16], were investigated, and remarkable improvements of their thermoelectric properties under pressure were observed. For transition metal dichalcogenides, the electronic properties of single-layer and multilayer MoS_2_ under pressure were analysed [17] and the effect of high pressure on the electronic and transport properties of 2H-MoS_2_ was also systematically studied [18]. Additionally, the thermoelectric properties of bulk SeS_2_ under hydrostatic pressure were investigated and an enhancement about 3.8 times of PF in the *c* direction at 20 GPa was observed [19]. For SnSe_2_, the crystal structure of bulk SnSe_2_ under pressure was studied by experiment [20]. As for layered transition-metal dichalcogenides, the figure of merit of these compounds is limited by the moderate PF due to their intrinsically low electronic conductivity [21], suggesting that a strategy to enhance thermoelectric performance *ZT* of layered transition-metal dichalcogenides is to increase their electrical transport properties. Recently, it has been found that higher electrical conductivity can be obtained for the monolayer and bilayer transition metal dichalcogenides when compared with bulk materials, and such phenomenon can be explained by the valley degeneracy at the band edge [21]. On the other hand, the thermoelectric properties of *n*-type layered transition-metal dichalcogenides can be enhanced under either normal compressive strain or biaxial compressive strain [22]. Meanwhile, application of hydrostatic pressure can offer an effective path to realize the reduction of out-of-plane and in-plane lattice constants as well; thus, applying external pressure is expected to be a potential method for improving thermoelectric performance of two-dimensional layered transition-metal dichalcogenides. Owing to low-dimensional structure and application of hydrostatic pressure, the thermoelectric properties of few-layered SnSe_2_ can be enhanced; however, the theoretical predictions of few-layered SnSe_2_ under pressure are lacking. Therefore, it is necessary to understand how the high pressure affects the electronic structures and transport properties of monolayer and bilayer SnSe_2_, to evaluate the role of interlayer atomic interactions and to explore the origins of the improvement of the thermoelectric parameters under pressure.

In this paper, the effect of pressure on the electronic and transport properties of monolayer and bilayer SnSe_2_ was systemically investigated by using first-principle calculations and semi-classic Boltzmann transport theory. The relationships among lattice parameters, electronic structures and thermoelectric properties of monolayer and bilayer SnSe_2_ under pressure were estimated, and the nature of difference in electronic conductivity of monolayer and bilayer SnSe_2_ under pressure was analysed. According to the doping dependence of electrical transport properties, we estimated the changes of PF of monolayer and bilayer SnSe_2_ with the increasing pressures. It is expected that the thermoelectric performance of few-layered SnSe_2_ can be effectively improved by applying external hydrostatic pressure.

## 2. Computational methods

**Figure 1.**
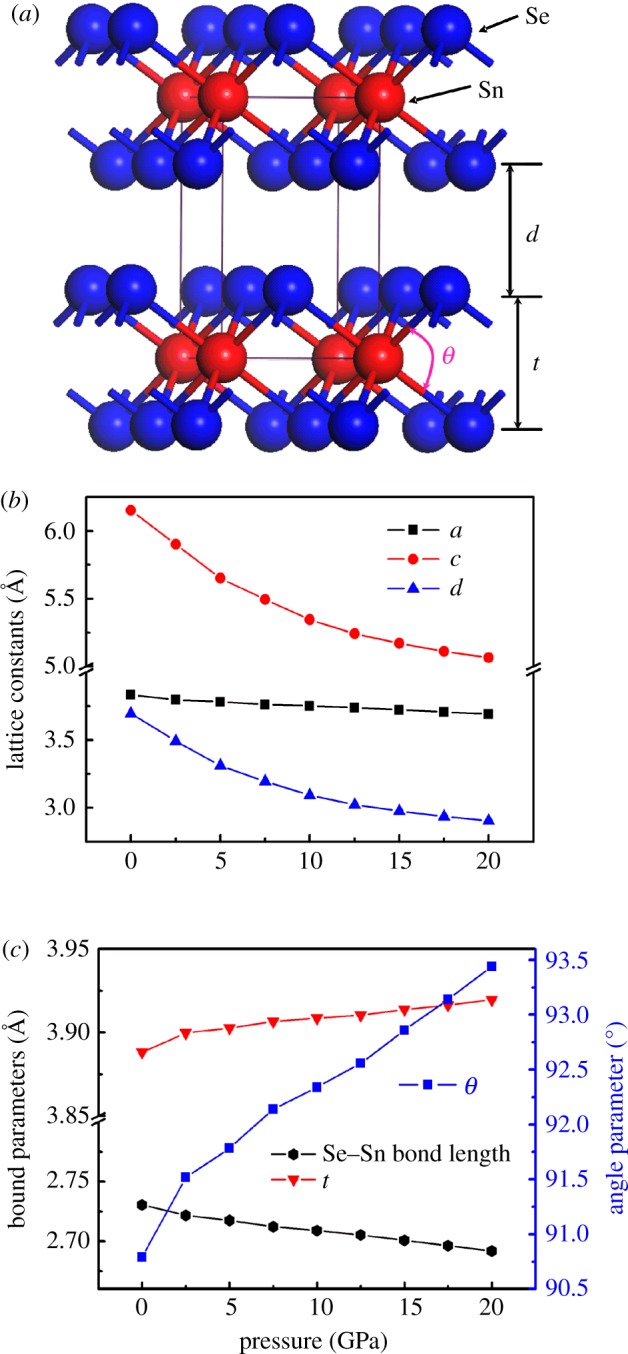
(*a*) Structure of bulk SnSe_2_ and definition of the bond angle *θ* and Se–Se distance. (*b*) Calculated lattice constants of bulk SnSe_2_ under pressure. (*c*) Computed bond parameters and bond angles of bulk SnSe_2_ versus pressure.

All calculations of the structural and electronic properties were carried out by using the projector-augmented wave [23] methods, as implemented in Vienna *ab initio* Simulation Package (VASP) [24]. The exchange correlation energy has been calculated using generalized gradient approximation (GGA) in the Perdew−Burke−Ernzerhof (PBE) form [25]. The cutoff energy of the plane-wave expansion is chosen to be 400 eV for all the calculations. The convergent criterion was 10^−6^ eV for energy and 0.03 eV Å^−1^ for force. The bulk SnSe_2_ compound is crystallized in the space group P3m with a layered crystal structure, as shown in figure 1*a*. For the geometric and self-consistent calculations of bulk SnSe_2_, a well-converged Monkhorst–Pack *k*-point grid of 7 × 7 × 5 is used to sample the Brillouin zones. The interlayer Se and Se interactions in SnSe_2_ are dominated by vdW interactions, which are overestimated in the simple GGA method [26]. In order to describe the interlayer interactions well, the vdW corrections using Grimme's vdW-D2 method are included in our calculations [27]. To apply external (hydrostatic) pressure in bulk SnSe_2_, we chose to add external stress to stress tensor in VASP code [24,28]. The structural change of bulk SnSe_2_ at different pressures up to 20 GPa was investigated, and lattice parameters and atomic positions of bulk SnSe_2_ under pressures are shown in figure 1*b*,*c*. As shown in figure 1*b*, the calculated lattice constants *a* and *c* of bulk SnSe_2_ at zero pressure are 3.835 and 6.153 Å, respectively, which are very close to experimental data (*a* = 3.81 Å and *c* = 6.14 Å) [29], and this is because the method of vdW-D2 can provide an excellent description of the crystal structure of bulk SnSe_2_ [26,27]. Figure 1*b* presents the variation of lattice constants of bulk SnSe_2_ under different pressures. From 0 to 20 GPa, the lattice constants of bulk SnSe_2_ decrease with the increase of hydrostatic pressures. It can also be found that the in-plane lattice constant *a* is much more weakly affected by pressure than out-of-plane lattice constant *c*. Meanwhile, the layer thickness *d* decreases under pressure, reflecting the fact that interlayer van der Waals interactions change with pressure. In order to gain an insight into the change of intralayer atomic positions under pressure, the bond and angle parameters at different pressures are shown in figure 1*c*. As the pressure increases from 0 to 20 GPa, the length of Se–Sn bond is decreased while the distance of Se–Se increases slightly under pressure, resulting in increase of the band angle under pressure. These structural parameters of bulk SnSe_2_ under pressure are then used to construct single- and double-layer SnSe_2_ structures corresponding to different pressures, and this approach has been applied to obtain these structures of single-layer and multilayer MoS_2_ under pressure [17]. For each monolayer or bilayer SnSe_2_, a vacuum region of 15 Å is added in the direction normal to the layers to avoid the interaction between the periodic images. The Brillouin zone is sampled using a Monkhorst–Pack *k*-point mesh (13 × 13 × 1) for the geometry optimizations and self-consistent calculation.

The BoltzTraP package was employed to calculate transport properties of single-layer and bilayer SnSe_2_ under different pressures [30]. The principle of this code is to solve transport properties based on semi-classical Boltzmann theory in conjunction with rigid band and constant relaxation time approximations, and it can yield accurate results for various types of thermoelectric materials [14–16,18,19,31,32]. In order to gain well-converged transport quantities, we employ a finer 35 × 35 × 1 *k*-mesh to calculate the transport properties.

## 3. Results and discussion

### 3.1. SnSe_2_ monolayer under pressure

**Figure 2.**
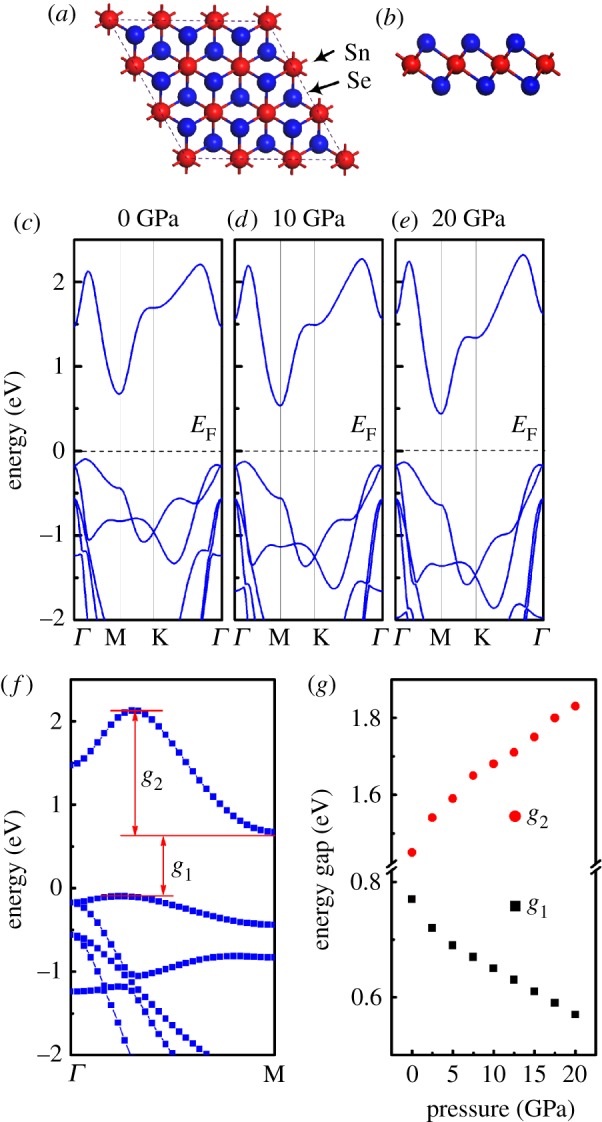
Top view (*a*) and side view (*b*) of monolayer SnSe_2_. (*c*–*e*) Electronic structure of monolayer SnSe_2_ under typical pressure of 0, 10 and 20 GPa, respectively. (*f*) Band structure of monolayer SnSe_2_ with pressure of 0 GPa. (*g*) Band gaps and change of band parameters of monolayer SnSe_2_ as a function of pressure.

As shown in figure 2*a,b*, the model of the SnSe_2_ monolayer can be viewed as cleaved from the SnSe_2_ surface, where a Sn atom is sandwiched between two Se atoms that formed a Se–Sn–Se triple layer. To investigate high pressure effect on the band of SnSe_2_ monolayer, hydrostatic pressures from 0 to 20 GPa were applied. The typical band structures of SnSe_2_ monolayer along the symmetry lines at different pressures are shown in figure 2*c–e*. At zero pressure, the band of SnSe_2_ monolayer shows an indirect band gap feature as reported [11]: the conduction band minimum (CBM) is located at M, while the valence band maximum (VBM) is located between *Γ* and M points. The calculated electronic band gap of SnSe_2_ monolayer at 0 GPa is 0.77 eV, which is slightly lower than the previous calculated value 0.85 eV [11], in which vdW corrections have not been considered. Additionally, we can see that an electronic pocket of the conduction band located between *Γ* and M points. The depth of the electronic pocket of the conduction band along the *Γ*−M direction near Fermi level can determine the electronic effective mass, and it then can determine the electrical conductivity of *n*-type SnSe_2_ monolayer, thus, it is a very important parameter (denoted as *g*_2_) for describing the band structure characteristic SnSe_2_ monolayer. With the increase of pressure from 0 to 20 GPa, the band gap of SnSe_2_ monolayer decreases almost linearly. On the other hand, the conduction band parameter *g*_2_ increases as pressure increases, which means the electron effective mass of SnSe_2_ monolayer becomes smaller with pressure. As discussed above, the reduction of conduction band effective mass brings out the decrease of the Seebeck coefficient along with the increase of the electrical conductivity of *n*-type thermoelectric materials [33–36]. Therefore, the increase of conduction band parameter *g*_2_ under pressure will cause change of the transport properties of *n*-type SnSe_2_ monolayer.

**Figure 3.**
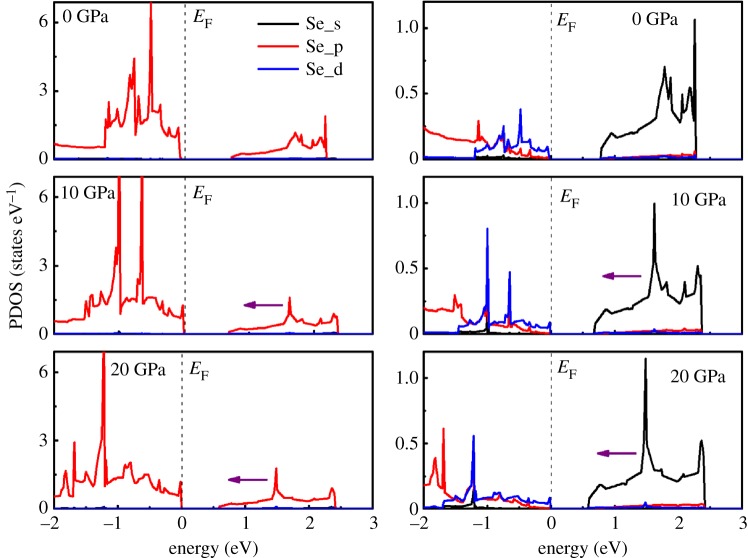
Calculated projected density of states of monolayer SnSe_2_ at different pressures. The Fermi levels are set to zero.

In order to further understand the electronic structure under pressure, the partial densities of states (PDOS) of monolayer SnSe_2_ in the energy interval between −2 eV and 3 eV at pressure of 0, 10 and 20 GPa are shown in figure 3. From the PDOS at zero pressure, we can see that the CBM of monolayer SnSe_2_ primarily comes from Se 4*p* and Sn 4*d* orbitals, whereas the VBM is dominated by the Se 4*p* orbital and Sn 5*p* and 4*d* orbitals. The characteristics of PDOS of CBM and VBM in monolayer SnSe_2_ are similar to bulk SnSe_2_ [37]. When the high pressure is applied, an obvious shift to the *E*_F_ of both the Sn 5*s* orbital and Se 4*p* orbital in the valence band in PDOS can be observed, resulting in the decrease of the band gap. On the other hand, the movement of Sn 5*s* orbital and Se 4*p* orbital in the valence band gradually getting close to the Fermi level under pressure is the reason of the change of conduction band parameter *g*_2_, which can determine the transport properties of *n*-type SnSe_2_ monolayer.

**Figure 4.**
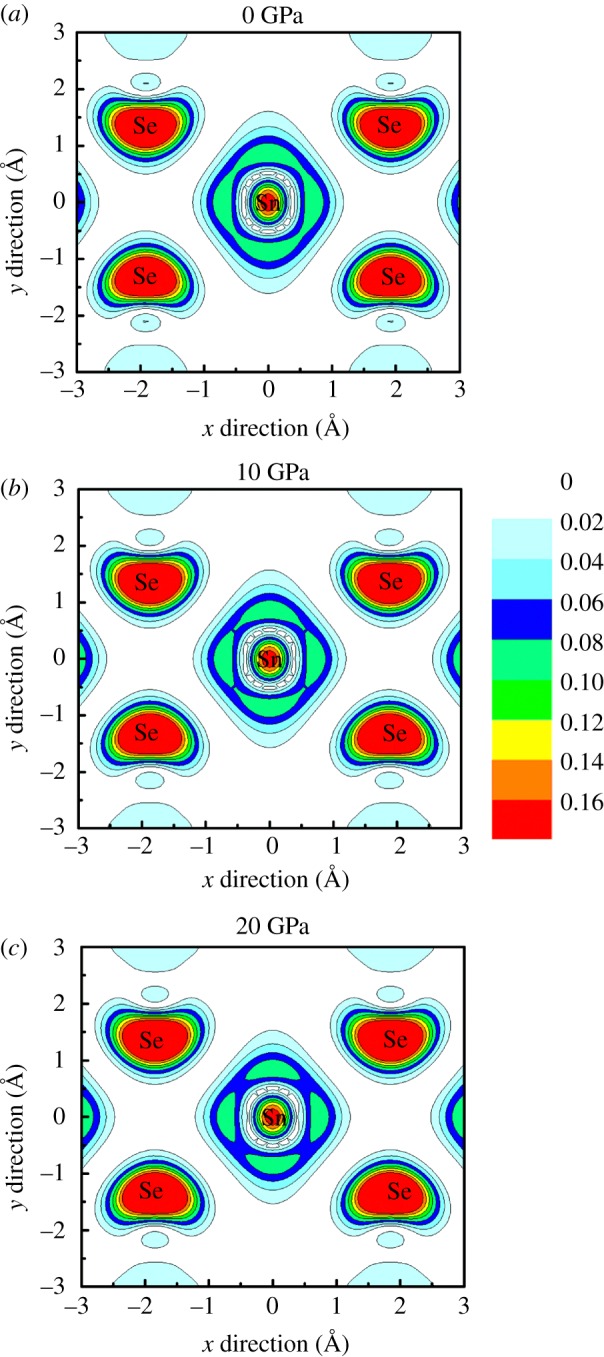
Contour plots of the partial charge density in the conduction band bottom at M point of monolayer SnSe_2_ on the Se–Sn–Se plane at different pressures. (*a*) 0 GPa, (*b*) 10 GPa and (c) 20 GPa. The Fermi levels are set to zero. The unit of charge density is e A^−3^.

In order to further understand the band engineering induced by hydrostatic pressure, the partial charge densities in the conduction band bottom at M point of monolayer SnSe_2_ under different pressures were plotted in figure 4. At free states without external pressure, the conduction band bottom at M point is mainly contributed by the Sn 5*s* orbital and Se 4*p* orbital according to the calculated PDOS, and there exist obvious antibonding characteristics between Se and Sn atoms. As seen from figure 4, the charges of Sn atom tends to escape from Sn atomic site which means the Sn 5*s* orbitals become delocalized with the increase of the external pressure; that is, the Se–Sn antibonding states become more hybridized with increasing pressure, which causes the electrical conductivity increase under external pressure. Such pressure-dependent distribution of charge density is also observed in other thermoelectric materials [14]. The charge delocalization of Sn 5*s* orbitals under pressure is consistent with the analysis from PDOS, and it can be ascribed to the change of Se–Sn bond length under pressure. With the increase of external pressure, the Se–Sn bond length decreases linearly, and then the overlapping of the Se and Sn atomic orbitals will rise, and it will drive the charges of Sn atom to gather close to the Sn atom.

**Figure 5.**
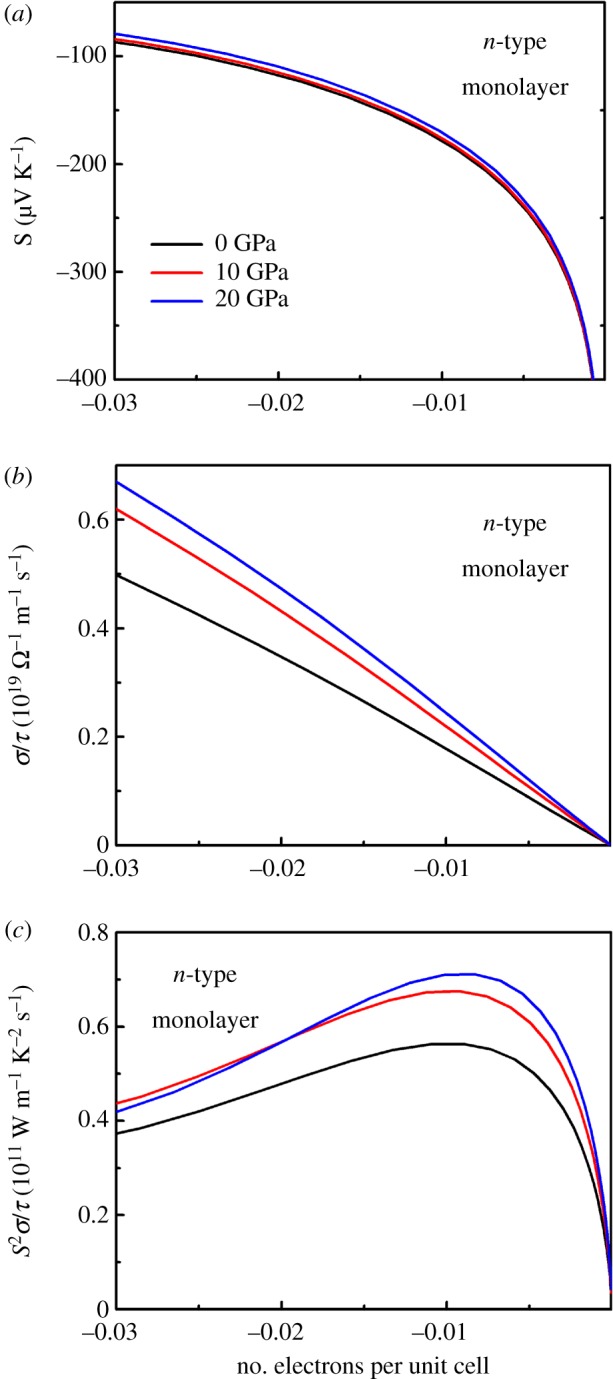
Calculated electronic transport coefficients of *n*-type monolayer SnSe_2_ at different pressures as a function of number of electrons per unit cell at 300 K. (*a*) Seebeck coefficient *S*, (*b*) electrical conductivity with respect to relaxation time *σ*/*τ* and (c) PFs with respect to relaxation time *S^2^σ/τ*.

We now turn our attention to assess the transport properties of monolayer SnSe_2_ under pressure. Since experimental works reveal that SnSe_2_ tends to form *n*-type semiconductors [33–36], we only considered the *n*-type doping in the following thermoelectric properties of monolayer or bilayer SnSe_2_. It is reported that the effect of spin–orbit interaction on the electronic structure of monolayer or bilayer SnSe_2_ is negligible [11]; therefore, the spin–orbit coupling effect is not included in layer-dependent studies of electronic transport properties in this work. In figure 5, we plot the results of *n*-type monolayer SnSe_2_ for the Seebeck coefficient, electrical conductivity and PFs at different pressures as a function of number of electrons per unit cell at room temperature. As can be seen in figure 5*a*, the Seebeck coefficient reduces slightly when applying external pressure, and such phenomenon is also observed in other thermoelectric systems under pressure [14,18,19]. As shown in figure 5*b*, the pattern of electrical conductivity with respect to relaxation time (*σ/τ*) change with pressure is just opposite: the *σ/τ* increases with the increase of pressure. The electrical conductivity *σ/τ* of *n*-type monolayer SnSe_2_ increases with pressure, which agrees with the above analysis of the PDOS and partial charge density of monolayer SnSe_2_ under pressure. Combining the values of Seebeck coefficient and electrical conductivity, we can obtain the PFs with respect to relaxation time (*S*^2^*σ*/*τ*). The results of *S*^2^*σ*/*τ* of *n*-type monolayer SnSe_2_ under pressure as a function of electrons per unit cell are shown in figure 5*c*. Comparing with the PFs at 0 GPa, the values of *S*^2^*σ*/*τ* increase under pressure in the whole range of carrier concentrations, and this is because the reduction of Seebeck coefficient is excessively compensated by the electrical conductivity under pressure. The PF *S*^2^*σ*/*τ* of *n*-type monolayer SnSe_2_ can be improved under pressure, indicating that the band structure manipulated by external pressure is a potent method to improve the thermoelectric properties of *n*-type monolayer SnSe_2_.

### 3.2 Bilayer SnSe_2_ under pressure

**Figure 6.**
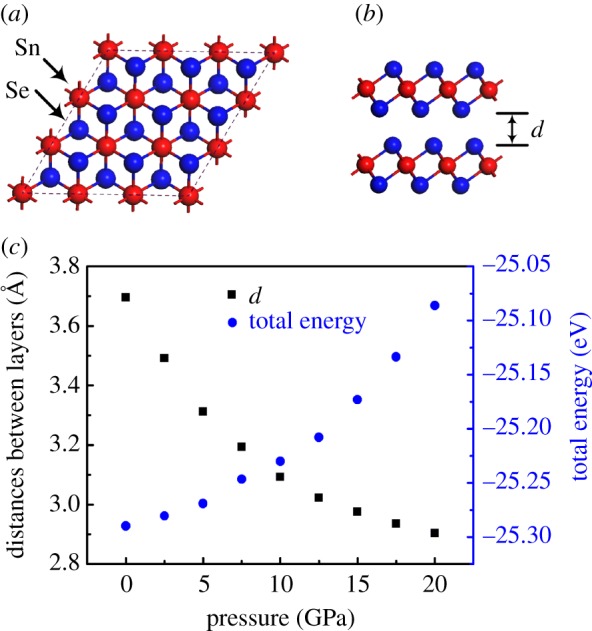
(*a*,*b*) Top and side view of bilayer SnSe_2_. (*c*) The distance between the layers and the total energy of bilayer SnSe_2_ as a function of pressure.

Previous sections have only discussed the monolayer SnSe_2_ for band structure and electronic transport properties under pressure. The effect of pressure on monolayer SnSe_2_ is primarily from changing the intralayer atomic interactions. In order to further investigate the influences of intralayer and interlayer atomic interactions of the layered SnSe_2_, the pressure-dependence of band structure and transport properties of bilayer SnSe_2_ under pressure are also discussed in this work. The top and side views of SnSe_2_ bilayer are shown in figure 6*a–b*, respectively. The distance between the layers and minimum total energy of bilayer SnSe_2_ at different pressures are shown in figure 6*c*. From 0 to 20 GPa, the distance between the layers decreases with the increase of hydrostatic pressures, which can result in the strengthening of interlayer van der Waals interactions under pressure. Meanwhile, we can see from figure 6*c* that the total energy of bilayer SnSe_2_ increases as the increase of pressure. The total energy of bilayer SnSe_2_ at zero pressure is the minimum, which means the structure under the free state is the most stable. With the pressure increasing, the minimum total energy of bilayer SnSe_2_ increases, suggesting that the stable structure of bilayer SnSe_2_ at higher total energy can be developed under proper geometrical constraint.

**Figure 7.**
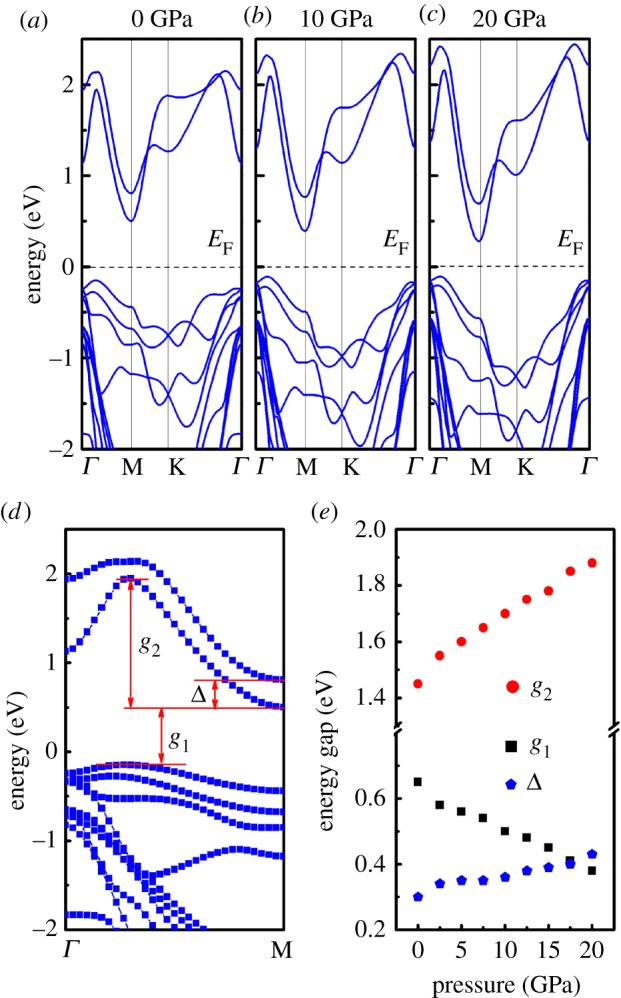
(*a*–*c*) Band structures of bilayer SnSe_2_ under pressure for 0, 10 and 20 GPa, respectively. (*d*) Partial band structure of bilayer SnSe_2_ at 0 GPa. (*e*) Band gaps and change of band parameters of bilayer SnSe_2_ as a function of pressure.

Figure 7*a*–*c* presents the band structures of bilayer SnSe_2_ for 0, 10 and 20 GPa. From the band structure at 0 GPa, an indirect gap of 0.65 eV can be obtained, which is slightly less than the one (0.77 eV) of the monolayer SnSe_2_. The layer-dependent band gap is observed in SnSe_2_ and other two-dimensional transition metal dichalcogenide materials [22,26]. As in the case of bilayer SnSe_2_, the CBM is located at M point while the VBM is located between *Γ* and M points, and there are pockets in the valence band. Figure 7*a–c*, displays the calculated band structure of bilayer SnSe_2_ under different pressures. It is found that the band gap decreases under pressure and the pockets of the valence band change with pressure. Meanwhile, the conduction band structure of bilayer SnSe_2_ contains two pairs of parabolic bands which are different with monolayer SnSe_2_. The important band parameters of bilayer SnSe_2_ are indicated in figure 7*d* and then summarized in figure 7*e*. Similar to the monolayer SnSe_2_, the pocket parameter of conduction band (*g*_2_) increases almost linearly. Additionally, there is a parameter *Δ* which represents the width of valence band splitting, and it grows linearly as the pressure increases.

**Figure 8.**
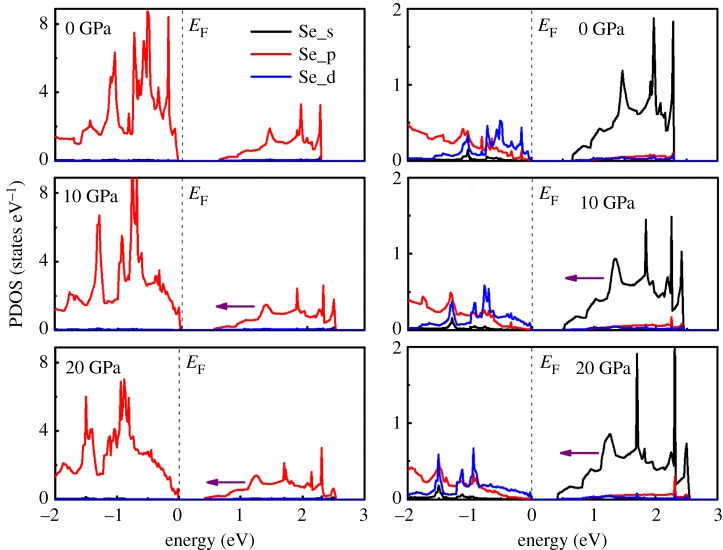
Projected density of states of bilayer SnSe_2_ under pressure of 0, 10 and 20 GPa. The violet arrow denotes the moving direction of peaks. The Fermi levels are set to zero.

To gain a deeper insight of the pressure-dependent electronic structure of bilayer SnSe_2_, the projected density of states of bilayer SnSe_2_ at different pressures are plotted in figure 8. It reveals that the valence band of bilayer SnSe_2_ consists of Se 4*p* and Sn 5*s* orbitals, and the energy states near Fermi level are also affected by external pressure: the peaks of Se 4*p* and Sn 5*s* orbitals move toward the Fermi level with the increase of pressure. This tendency is in accordance with the PDOS of monolayer under pressure. The variation of energy states near Fermi level can determine the transport properties of bilayer SnSe_2_ under pressure.

**Figure 9.**
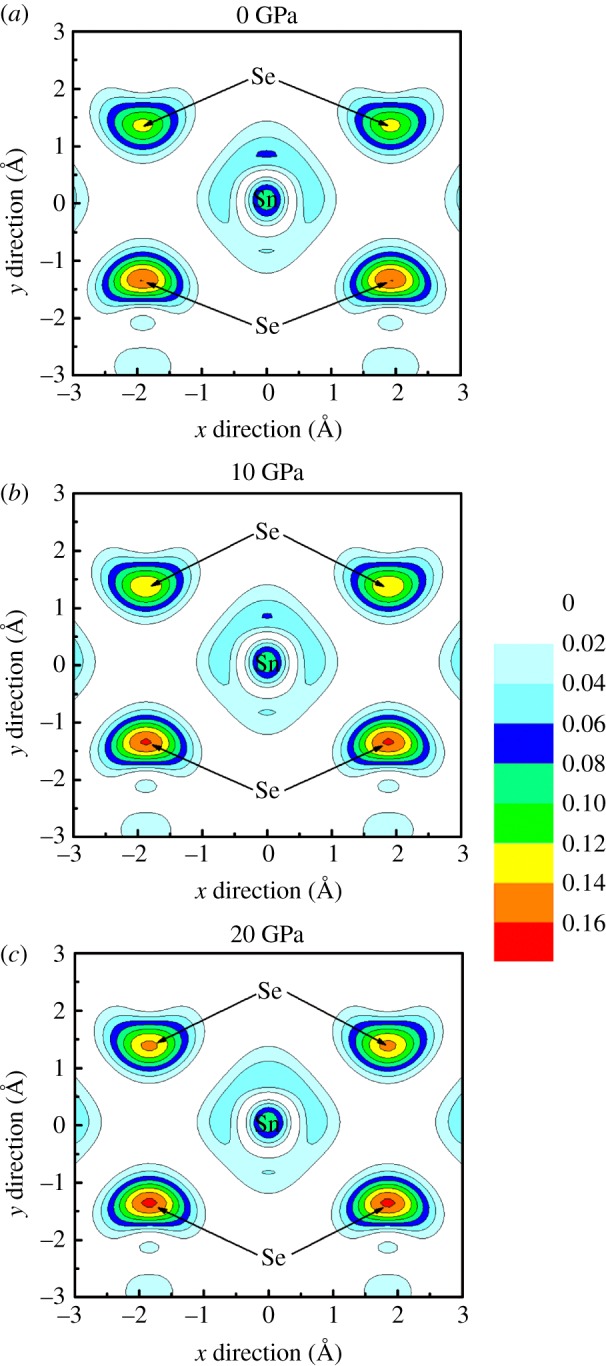
Contour plots of the partial charge density in the CBM at M point of monolayer SnSe_2_ on the Se–Sn–Se plane. (*a*) 0 GPa, (*b*) 10 GPa and (*c*) 20 GPa. The Fermi levels are set to zero. The unit of charge density is e A^−3^.

In order to further explore the subtle differences of electronic states between bilayer and monolayer SnSe_2_, the partial charge densities corresponding to CBM of bilayer SnSe_2_ are shown in figure 9. At zero pressure, the charge densities around Se and Sn atoms of bilayer SnSe_2_ are less than the monolayer one, which means the coupling of the Se–Sn antibonding states in bilayer SnSe_2_ becomes weaker than monolayer SnSe_2_, resulting in bilayer SnSe_2_ possessing less electrical conductivity than monolayer. In view of the construction methods of few-layered SnSe_2_, the intralayer atomic positions of bilayer and monolayer SnSe_2_ are the same, and the intralayer atoms have the same impact on the charge density distribution. Thus, the decrease of charge densities in bilayer SnSe_2_ compared to monolayer can be ascribed to the presence of interlayer atomic interactions: the charge densities around Sn and Se tend to gather in the direction of adjacent layer in bilayer SnSe_2_ due to the coupling of interlayer introduced by vdW interactions. Meanwhile, another obvious feature is that the charge densities distribution is asymmetric in upper and lower parts, as shown in figure 9*a*, and this is because the upper two atoms are close to the vacuum region while the lower two atoms are close to the adjacent layer, resulting in the charge redistribution induced by layers coupling. With the increase of pressure, the charge densities around Se atoms become denser (figure 9), suggesting the Se–Sn antibonding states become more hybridized with increasing pressure, and it will enhance the electrical conductivity of bilayer SnSe_2_ under pressure.

**Figure 10.**
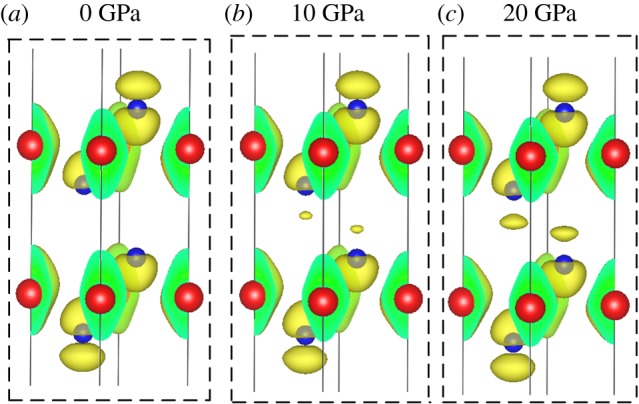
Isosurfaces of band-decomposed charge density at M point of the conduction band bottom of bilayer SnSe_2_ at different pressures. (*a*) 0 GPa, (*b*) 10 GPa and (*c*) 20 GPa. The red and blue balls represent Sn and Se atoms, respectively.

As a further support, the band-decomposed charge density at M point of CBM in bilayer SnSe_2_ is shown in figure 10. It is found that there emerges an increasing charge distribution between the layers as pressure increases, and it can be ascribed to shortening of the layer thickness *d* under pressure (figure 1*b*), reflecting the fact that the layers' coupling strengthens, induced by interlayer van der Waals interactions under pressure.

**Figure 11.**
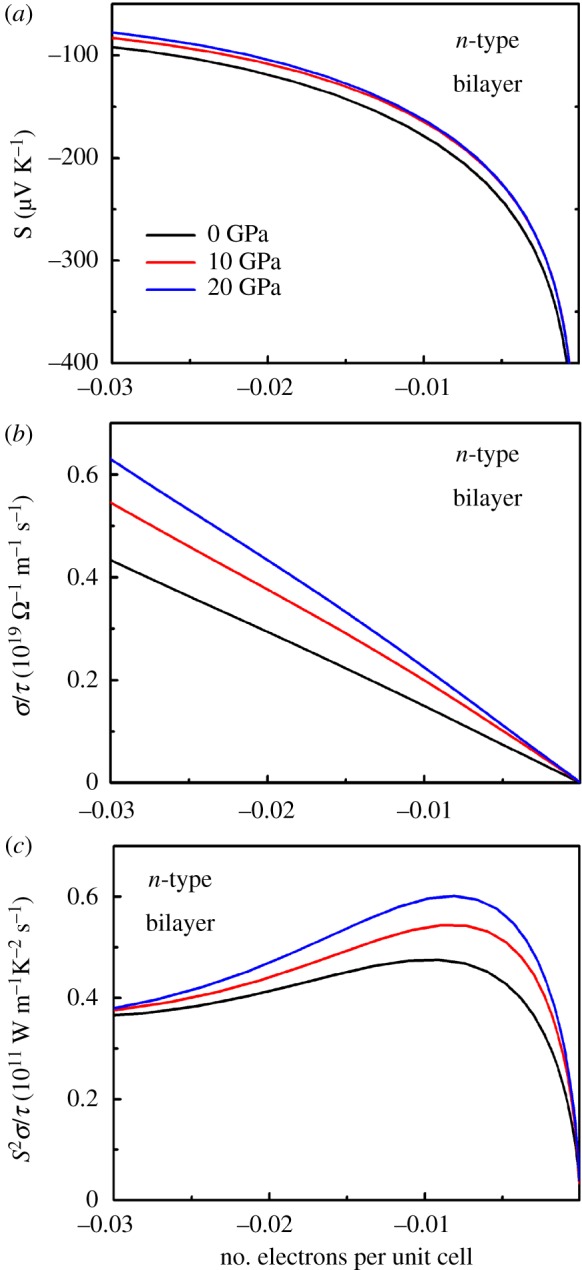
(*a*) Seebeck coefficient *S*, (*b*) electrical conductivity with respect to relaxation time *σ*/*τ*, (*c*) PFs with respect to relaxation time *S^2^σ/τ* of *n*-type bilayer SnSe_2_ under pressure for 0, 10 and 20 GPa.

Based on the electronic structure of bilayer SnSe_2_ under pressure, we now discuss the effect of pressure on the transport properties in bilayer SnSe_2_ based on semi-classical Boltzmann theory. In figure 11, we show the doping-dependent *S*, *σ*/*τ* and *S^2^σ/τ* of *n*-type bilayer SnSe_2_ under different pressures. From figure 11*a*, we can see that the Seebeck coefficient of bilayer SnSe_2_ has a slight decrease under pressure in the whole carrier concentration, and this tendency is consistent with monolayer SnSe_2_ under pressure. In figure 11*b*, we show the calculated electrical conductivity with respect to relaxation time *σ*/*τ* under pressure. We can see the value *σ*/*τ* of bilayer SnSe_2_ at zero pressure is 0.434 × 10^19^ $\Omega^{-1}\;m^{-1}\;s^{-1}$ at 3% *n* doping, which is slightly lower than monolayer SnSe_2_ (0.498 × 10^19^ $\Omega^{-1}\;m^{-1}\;s^{-1}$, and this is mainly caused by interlayer interactions in bilayer SnSe_2_, as we discussed above. As shown in figure 11*b*, electrical conductivity with respect to relaxation time *σ/τ* of bilayer SnSe_2_ increases with increasing pressure, and this is consistent with the previous analysis of the band structures and charge density distribution of bilayer SnSe_2_ under pressure. Figure 11*c* shows the calculated *S^2^σ/τ* of *n*-type bilayer SnSe_2_ under pressure. It can be observed that although the Seebeck coefficient decreases with pressure, the PF of *n*-type bilayer SnSe_2_ still increases because the electrical conductivity of the system is increasing [19]. The largest value of PF in *n*-type bilayer SnSe_2_ at all pressures is found at 20 GPa which is computed to be 0.60 × 10^11^ W K^−2^ m^−1^ s^−1^ at the optimal carrier concentration, while the maximum value at 0 GPa is only 0.47 × 10^11^ W K^−2^ m^−1^ s^−1^. Therefore, the PF of *n*-type bilayer SnSe_2_ increases by 27% by increasing the pressure to 20 GPa at 300 K. It is reported that the average PF of bulk SnS_2_ can increase by 33% by increasing the pressure to 20 GPa at 800 K [19]. Bhattacharyya *et al.* [22] investigated the effect of strain on thermoelectric properties of few-layered MoS_2_, and the PF of *n*-type bilayered MoS_2_ at 900 K can be improved by 28% and 35% in the case of −8% normal compressive strain and biaxial compressive strain, respectively. Compared with these layered materials, it can be found that the enhancing of the pressure-dependent PF for *n*-type bilayer SnSe_2_ is significant, suggesting that it can provide a useful guide for improving the thermoelectric performance of few-layered SnSe_2_ through manipulating lattice strain induced by pressure.

## 4. Conclusion

In summary, we carried out first-principles calculations to investigate the effect of high pressure on the electronic structure and thermoelectric performance of monolayer and bilayer SnSe_2_. Results show that the *n*-doping electrical conductivity of monolayer and bilayer SnSe_2_ can be improved under pressure, and it is attributed to pressure-induced changes of the Se–Sn antibonding states in conduction band. Meanwhile, the discrepancy of transport properties in monolayer and bilayer SnSe_2_ was estimated, and monolayer SnSe_2_ is observed to possess higher electrical conductivity, and the electrical conductivity of monolayer SnSe_2_ is only controlled by intralayer atomic interactions while that of the bilayer SnSe_2_ is dominated by intralayer and interlayer atomic interactions. Our results clearly demonstrate that the PF of both monolayer and bilayer SnSe_2_ can be improved by applying external pressure. This research can be useful to understand the origins of the transport properties for monolayer and bilayer SnSe_2_ under pressure, and it offers valuable insight of the lattice strain induced by pressure as an efficient route for improving the thermoelectric performance of few-layered SnSe_2_.

## Data accessibility

All data to reproduce figures are available on Dryad (http://dx.doi.org/10.5061/dryad.v6d9t) [38].
